# Mindfulness-based interventions for competitive anxiety in athletes: the moderating role of control type—Preliminary evidence from a systematic review and meta-analysis

**DOI:** 10.3389/fpsyg.2026.1832397

**Published:** 2026-05-28

**Authors:** Kaiming Chen, Yiran Xing

**Affiliations:** 1 School of Arts and Social Sciences, The University of Sydney, Sydney, NSW, Australia; 2 Department of Physical Education, Anhui University, Hefei, China

**Keywords:** athletes, competitive anxiety, control type, meta-analysis, mindfulness

## Abstract

This meta-analysis examined the effects of mindfulness-based interventions (MBI) on competitive anxiety in athletes. Eighteen controlled trials (*N* = 667) from seven databases were included. Random-effects modeling revealed a moderate overall effect favoring MBI [*g* = −0.53, 95% CI (−0.74, −0.32), *I*^2^ = 63%], with greater reductions in cognitive anxiety (*g* = −0.58) than somatic anxiety (*g* = −0.41). Control type was the only statistically significant moderator (*p* = 0.005): MBI showed moderate effects vs. waitlist controls (*g* = −0.66) but near-zero effects vs. active controls (*g* = −0.05). Total intervention hours did not significantly predict effect size (β = −0.025, *p* = 0.062). Trim-and-fill adjustment yielded *g* = −0.44. Because the active-control stratum contained only three comparisons, the near-zero estimate against active comparators should be treated as preliminary. The observed pattern is nevertheless compatible with the interpretation that the anxiety reduction attributable to MBI reflects, to a substantial extent, nonspecific intervention factors rather than mindfulness-specific mechanisms.

## 1 Introduction

Competitive anxiety is one of the most widely studied psychological constructs in sports psychology, directly impacting athletic performance and athletes' mental health. The multidimensional competitive anxiety theory developed by Martens et al. (1990) conceptualizes the construct as three conceptually distinct but empirically correlated dimensions, namely cognitive anxiety (worry and disruptive thought about performance), somatic anxiety (perceived physiological arousal), and self-confidence (subjective expectancy of successful performance). Each dimension is held to follow its own temporal trajectory prior to competition and to exert partly independent effects on performance, and the Competitive State Anxiety Inventory-2 (CSAI-2) was developed as the operational instrument anchoring this framework, laying the theoretical and methodological foundation for this field. Theoretical explanations regarding the relationship between anxiety and performance have evolved from the inverted U-shaped hypothesis to the catastrophe model (Hardy et al., 2018) and then to attentional control theory (Ford et al., 2017), reflecting a deepening understanding of the mechanisms by which anxiety affects athletes. Epidemiological syntheses indicate that anxiety and depression symptoms are common among current elite athletes, with pooled prevalence estimates around 34% (Gouttebarge et al., 2019). Clinical anxiety of this kind is conceptually distinct from situation-specific competitive anxiety, which refers to time-bound cognitive and somatic responses surrounding competition (Martens et al., 1990), and the two constructs are not treated as interchangeable in the present synthesis. From a longitudinal perspective, persistent competitive anxiety is closely associated with burnout (Lin et al., 2022; Glandorf et al., 2025) and premature withdrawal from athletic careers (Battaglia et al., 2024; Zhang et al., 2024), constituting a significant psychological risk factor threatening athletes' long-term development. Therefore, exploring effective anxiety management strategies has both theoretical and practical significance. An unresolved question, and the one that motivates the present synthesis, concerns whether the anxiety-reducing influence of mindfulness-based interventions arises from mechanisms unique to mindfulness practice or from factors common to most structured psychological interventions delivered to athletes.

In recent years, MBI have gradually become a research hotspot in the field of sports psychology due to their unique mechanisms in attention regulation and emotional acceptance. Mindfulness is defined as purposeful, non-judgmental attention to the present experience (Kabat-Zinn, 2009). Its core anxiety-reducing mechanism lies in reducing rumination on threatening cognitions through awareness and acceptance, rather than attempting to suppress or eliminate negative internal states (Birrer et al., 2012). Cross-sectional evidence from 299 elite Turkish male footballers indicates that dispositional sport mindfulness moderates the relationship between pre-competition threat appraisal and negative emotional states (Solmaz and Yarayan, 2025), and a complementary study with 363 student-athletes reports that depression, anxiety, and stress partially mediate the association between sport mindfulness and self-rated performance (Tingaz et al., 2023). Unlike traditional mental skills training that directly changes negative thinking, mindfulness interventions aim to change the relationship between individuals and their physical and mental states (Pineau et al., 2014). Several structured mindfulness programmes have been developed for the sport context, including Mindfulness-Acceptance-Commitment Training (MAC; Gardner and Moore, 2004), the Mindful Sport Performance Enhancement programme (MSPE; Kaufman et al., 2009, 2020), Mindfulness-Acceptance-Insight-Commitment Training (MAIC; Su et al., 2024), adaptations of Mindfulness-Based Stress Reduction (MBSR), and Mindfulness Meditation Training for Sport [MMTS 2.0 (Cambridge University Press, Cambridge, United Kingdom); Baltzell, 2016]. Among these programmes, MAIC extends the acceptance-commitment architecture common to MAC by inserting an explicit insight module, in which athletes are trained to recognize recurrent self-referential thought patterns, to reappraise their functional role during competition, and to integrate this metacognitive awareness with values-based behavioral commitments. This insight component is positioned as the principal theoretical differentiator between MAIC and other manualised mindfulness protocols. Differences in theoretical orientation across programmes may translate into differences in effectiveness, and comparative cross-cultural evidence has pointed to the potential value of culturally-adapted protocols (Du and Ning, 2024). Meanwhile, traditional methods such as progressive muscle relaxation and cognitive behavioral therapy also have considerable evidence in anxiety relief (Lange-Smith et al., 2024), making it a crucial question whether mindfulness interventions have specific effects beyond general intervention factors.

Several meta-analyses have examined the effects of mindfulness interventions on athletes' anxiety or performance, but methodological improvements are possible. Wang et al. (2024) searched five databases up to July 2023, including 20 studies, and reported a pooled effect size of MBI for competition anxiety, *g* = −0.67 [95% CI (−0.92, −0.42)]. That synthesis relied on the PEDro scale for risk-of-bias appraisal, a choice suited to physiotherapy trials but less well aligned with the Cochrane RoB 2.0 (Cochrane, London, United Kingdom) tool recommended for randomized trials of behavioral interventions, given the moderate agreement between the two tools on domains most relevant to behavioral trials (Armijo-Olivo et al., 2015). More importantly, this study mixed analysis of general anxiety scales (BAI, STAI, DASS, BSI) with sports-specific scales, blurring the boundaries of the situation-specific construct of competition anxiety, and included progressive muscle relaxation training from Liang et al. (2021)—a non-mindfulness intervention. Zhang et al. (2025) retrieved data up to October 2024, including 32 RCTs (*N* = 1,108), to examine the effects of mindfulness training on various psychological states in elite athletes. They found that MBSP and MT could alleviate anxiety, and MAIC could alleviate mental fatigue. However, this study covered a wide range of psychological dimensions rather than focusing on a multidimensional analysis of competitive anxiety, and did not distinguish between control groups. Li et al. (2025) examined the effects of psychological interventions on athlete anxiety more broadly, including 24 studies (*N* = 853). They found that the overall effect size of psychological interventions was large (SMD = −0.99), and the effect size of mindfulness intervention in subgroups was SMD = −0.55, lower than that of mental skills training (−1.20) and cognitive behavioral therapy (−0.85). This finding suggests that mindfulness may not be the optimal choice for anxiety management, but direct head-to-head comparisons are needed to verify this. Si et al. (2024) focused on the intervention effects of MBI on athlete performance, finding that mindfulness training significantly improved athletic performance and psychological indicators, but the anxiety-related analysis was limited. The common limitations of the aforementioned studies are: they all only conducted subgroup analyses without using meta-regression to test the linear trend of continuous variables, their publication bias tests were insufficient, and most importantly, they all failed to systematically distinguish between control types—a distinction that determines whether the MBI effect stems from a mindfulness-specific mechanism or a non-specific intervention factor.

Based on this review, existing research provides room for updating this meta-analysis in several aspects. Regarding timeliness, the search results only extend to July 2023 (Wang et al., 2024) or October 2024 (Zhang et al., 2025). Newer studies such as those by Yu et al. (2024), Gan et al. (2024), Josefsson et al. (2024), and Kelemen et al. (2025) have been published since then, requiring the inclusion of updated evidence. Regarding quality assessment, the PEDro scale is unsuitable for assessing the risk of bias in RCTs; the Cochrane RoB 2.0 (Sterne et al., 2019) should be used instead. Regarding the testing of moderating variables, continuous variables such as total intervention duration and sample size require meta-regression rather than simple dichotomous subgrouping. In terms of distinguishing control types, the difference in effect size between waitlist controls and active controls has significant theoretical implications—it determines whether the effect of MBI stems from its specific components (cognitive dissociation, acceptance attitude) or from non-specific factors (attention, social interaction, expectation effect). Existing head-to-head comparative studies (Hut et al., 2023; Dana et al., 2022; Tóth et al., 2023) have not found MBI to be superior to active controls, but this scattered evidence has not yet been systematically quantified. This aligns with the classic debate in clinical psychology regarding common and specific factors (Michopoulos et al., 2021; Cuijpers et al., 2024). Regarding construct purity, the mixed analysis of general anxiety scales and sports-specific scales, along with the inclusion of non-MBI interventions, blurs the dual boundaries between the competition anxiety construct and the intervention construct. This study strictly limited the use of sports-specific competition anxiety scales (CSAI-2/CSAI-2R, SAS-2, SCAT, CAI-T) to ensure construct validity (Putra and Guntoro, 2022; Tomczak et al., 2022; Trpkovici et al., 2023; Zhang et al., 2024, 2023).

Based on the above analysis, this study proposes the following research questions: (RQ1) What is the overall effect size of the MBI on athletes' competition anxiety (cognitive anxiety, somatic anxiety, self-confidence)? (RQ2) Do MBI type, sports type, athletic level, control type, anxiety scale type, and cultural background moderate the intervention effect? (RQ3) Is there a linear association between the total intervention duration, number of intervention weeks, sample size, year of publication, and mean age and the effect size? (RQ4) Is there publication bias in the evidence included in the studies?

## 2 Methods

### 2.1 Inclusion criteria and search strategy

Inclusion criteria were established using the PICOS framework. Eligible participants were athletes without pre-specified upper or lower age limits and at any competitive level from local amateur through to national standing, provided they were not engaged solely in recreational physical activity and were not receiving rehabilitation for injury or illness. The sample that ultimately satisfied all criteria comprised adolescent and adult athletes with study-level mean ages from 17.1 to 39.8 years, so the present synthesis does not address pre-adolescent children or older athletes beyond middle adulthood, and inferences are bounded accordingly. The intervention was required to be a structured mindfulness programme, operationally defined as a multi-session protocol of pre-specified content, session sequencing, and duration, delivered by a trained facilitator according to a written manual or otherwise reproducible description, and belonging to one of the established mindfulness families (MAC, MSPE, MAIC, adapted MBSR, MMTS 2.0, or structurally analogous variants). An intervention was considered mindfulness-centered when at least half of the cumulative protocol time was devoted to mindfulness exercises specified *a priori* as formal meditation, body-scan practice, present-moment attention training, awareness-of-thought practice, or acceptance-based experiential exercises, as documented in the intervention description of each primary report. Interventions in which such practices occupied less than half of protocol time, stand-alone yoga or Tai Chi programmes, and psychological interventions without a core mindfulness component were excluded. The control group consisted of a waitlist/no-treatment control or an active control (PST/psychological skills training/REBT/CBT/relaxation training; Röthlin et al., 2020; Gross et al., 2018; Turner, 2022; Jordana et al., 2023). Outcome measures were strictly limited to sports-specific competition anxiety scales: CSAI-2/CSAI-2R, SAS-2, SCAT, CAI-T, and the Acceptance State and Trait Sports Anxiety Scale, consistent with existing meta-analyses in the same field (Wang et al., 2024; Scott-Hamilton et al., 2016). Studies using only general anxiety scales (GAD-7, STAI, BAI, DASS, etc.) were excluded. The study design must be an RCT or a quasi-experimental design with a control group; case studies, cross-sectional studies, qualitative studies, and studies without control groups were excluded.

The theoretical basis for excluding general anxiety scales is that competition anxiety is a situation-specific multidimensional construct (Martens et al., 1990), fundamentally different from general anxiety in its theoretical basis and measurement dimensions (Mesagno et al., 2024). The decision to accept SAS-2 as a trait-level instrument rests on the partial construct overlap between SAS-2 and CSAI-2. Both instruments share cognitive-worry and somatic-arousal subcomponents, while SAS-2 adds a concentration-disruption subscale and CSAI-2 adds a self-confidence subscale, and the two differ primarily in temporal reference (stable tendency vs. situational state). The concurrent inclusion of state and trait instruments is therefore a source of residual construct heterogeneity, which is acknowledged in the Limitations; the dimensional analyses reported below restrict themselves to the subcomponents that are shared across instruments (Tang et al., 2023), and a sensitivity analysis restricted to state-scale studies is reported alongside the primary estimate.

Seven databases (PubMed, Web of Science, Scopus, PsycINFO, SPORTDiscus, CNKI, and Cochrane Library) were searched, spanning from inception to October 2025. The complete search strategy for PubMed comprised the three conceptual blocks (mindfulness OR mindfulness-based OR MBSR OR MAC OR MSPE OR MAIC OR meditation) AND (athlete^*^ OR sport^*^ OR player^*^ OR competitor^*^) AND (anxiety OR anxious OR “competitive anxiety” OR “sport anxiety”). Search strategies for the remaining six databases were adapted from this template, preserving the three conceptual blocks while accommodating each platform's syntax and controlled vocabulary. For Web of Science and Scopus, the equivalent TS and TITLE-ABS-KEY field codes were used with identical free-text terms; for PsycINFO and SPORTDiscus, corresponding thesaurus terms (for example Meditation, Sports Psychology, and Anxiety) were combined with the free-text string; for the Cochrane Library, the CENTRAL register was searched using the same Boolean structure. For CNKI, the three conceptual blocks were rendered in Simplified Chinese as (正念 OR 正念训练 OR 正念冥想 OR MBSR OR MAC OR MSPE OR MAIC OR 冥想) AND (运动员 OR 运动 OR 选手) AND (焦虑 OR 竞赛焦虑 OR 比赛焦虑 OR 运动焦虑). The per-database hit counts totalled 2,410 records, consistent with the PRISMA flow diagram; verbatim search strings, dates of last search, and per-database hit counts for all seven databases are provided in Supplementary Data Sheet 1. Supplementary searches—comprising forward and backward citation tracking of the included studies, hand-searching reference lists of four prior meta-analyses (Wang et al., 2024; Zhang et al., 2025; Li et al., 2025; Si et al., 2024), and gray-literature searching via ProQuest Dissertations and Theses Global—yielded an additional 35 records, bringing the identification-stage total to 2,445 records. Two three-arm studies (Dana et al., 2022; Tóth et al., 2023) contributed both a waitlist and an active comparator arm. Following Chapter 23 of the Cochrane Handbook (Higgins et al., 2024) and the worked tutorial by Axon et al. (2023), two distinct analytic procedures were applied. For the overall synthesis, each three-arm study was reduced to a single study-level effect size by combining its two control arms using the Chapter 23 weighted-mean formula, yielding *k* = 18. For the control-type moderator analysis, in which the contrast between mindfulness-based intervention and each specific control type is the quantity of interest, the three-arm studies contributed two separate comparisons apiece (*k* = 20), with the MBI sample apportioned between the two comparisons and the variance of each split comparison calculated in accordance with Chapter 23. The Cochrane Handbook notes that the split-sample approach only partially addresses the unit-of-analysis issue because the resulting comparisons remain correlated; the approach is nonetheless appropriate when investigating intervention-related sources of heterogeneity, as is the case for the present control-type contrast, and sensitivity analyses examined the robustness of the control-type contrast to alternative handling of the three-arm studies. Two researchers independently screened titles and abstracts against the eligibility criteria, with records flagged by either reviewer as potentially relevant advancing to full-text assessment. Full-text eligibility decisions were likewise performed in duplicate, and inter-rater agreement was monitored throughout. Disagreements were resolved through discussion between the two reviewers and, where consensus could not be reached, adjudicated by a third researcher. Data extraction followed a pre-piloted coding sheet that captured the following fields for each study: (a) study identification (first author, year, country); (b) participant characteristics (*N* per arm, mean age, sex distribution, sport, competitive level); (c) intervention parameters (mindfulness-programme family, total intervention hours, number of weeks, number of sessions, session length); (*d*) comparator characteristics (waitlist/no-intervention vs. active comparator, and the specific modality of any active comparator); (e) outcome-measurement characteristics (instrument, subscale, time-point); and (f) effect-size data (pre- and post-intervention means and standard deviations per arm, or *F*/η*p*^2^ statistics when descriptive statistics were unavailable). Risk-of-bias ratings on each of the five Cochrane RoB 2.0 domains were recorded alongside the extraction fields. Extraction was performed in duplicate, and classification of mindfulness-based-intervention family and of comparator type was anchored in the authors' own intervention descriptions in each primary report, with ambiguous cases flagged for discussion and the resolution recorded.

### 2.2 Risk of bias assessment

The Cochrane RoB 2.0 (Sterne et al., 2019) was used instead of the PEDro scale. Two researchers independently assessed five domains: D1 randomization process, D2 deviation from the predetermined intervention, D3 missing outcome data, D4 outcome measurement, and D5 selective reporting. Cohen's Kappa was calculated to measure inter-rater consistency, and visualizations were generated using the R package robvis.

### 2.3 Data analysis

Analysis was performed using the R software package metafor (v4.4; R Foundation for Statistical Computing, Vienna, Austria) (Harrer et al., 2021). Hedges' *g* (corrected for small sample bias) was used as the effect size indicator; a negative value indicated lower anxiety in the intervention group than in the control group. A REML random-effects model was used to estimate the pooled effect (Borenstein et al., 2021). Heterogeneity was assessed using Cochrane's *Q* and *I*^2^, with effect size benchmarks based on Cohen (2013): 0.2 for a small effect, 0.5 for a medium effect, and 0.8 for a large effect. Effect size calculations preferentially employed the post-test between-group difference method. For the minority of studies that reported only F-statistics or partial eta-squared without raw means and change-score standard deviations, the change-score standard deviation was derived from the MS_error term of the repeated-measures analysis of variance, and *g* was computed using the change-score formula set out by Chandler et al. (2019). This transformation rests on the assumptions of homogeneity of variance across groups and sphericity of the repeated-measures covariance structure. This transformation was applied to three of the eighteen included studies that reported only F-statistics or partial eta-squared without raw means and standard deviations. A sensitivity analysis excluding these three studies yielded a pooled effect of *g* = −0.51 [95% CI (−0.73, −0.29), *I*^2^ = 61%], essentially unchanged from the primary estimate, indicating that the pooled finding is robust to possible departures from the homogeneity and sphericity assumptions. Residual conversion error cannot be fully excluded and is acknowledged in the Limitations. For studies using multidimensional scales (CSAI-2/2R, SAS-2), effect sizes were calculated separately for the three sub-dimensions of cognitive anxiety, somatic anxiety, and self-confidence, and then pooled independently.

Subgroup analyses were conducted for strata containing at least three comparisons, covering the type of mindfulness-based intervention, sport type, competitive level, control type, scale type (state vs. trait), and cultural background. Cultural background was operationalized using Hofstede's individualism–collectivism dimension (Hofstede, 2011; Hofstede et al., 2010), because this dimension has the most direct conceptual link to acceptance-based and self-referential processes implicated in mindfulness interventions, and because a theoretically anchored metric is preferable to a simple geographical dichotomy. Countries with an individualism index below 50 were classified as collectivist (China IDV = 20, Iran IDV = 41, Indonesia IDV = 14, Peru IDV = 16) and countries with an index of 50 or above as individualist (United States IDV = 91, Australia IDV = 90, Hungary IDV = 80, Germany IDV = 67, Spain IDV = 51, Sweden IDV = 71), following the cut-off convention commonly adopted in cross-cultural meta-analyses. Meta-regression was used to test the linear association between total intervention duration, number of intervention weeks, sample size, year of publication, and mean age and effect size. Publication bias was assessed using a four-step method: funnel plot visual inspection, Egger regression test, trim-and-fill correction, and fail-safe *N* (Bartoš et al., 2022, 2023; Borenstein, 2023). Sensitivity analyses included stepwise elimination, re-pooling after excluding studies with high risk of bias, testing robustness of conclusions after including studies on general anxiety scales, analyzing only state scale studies after excluding peculiar scales (*k* = 9), and analyzing only RCTs after excluding quasi-experimental designs (*k* = 12).

## 3 Results

### 3.1 Literature screening

**Figure 1:**
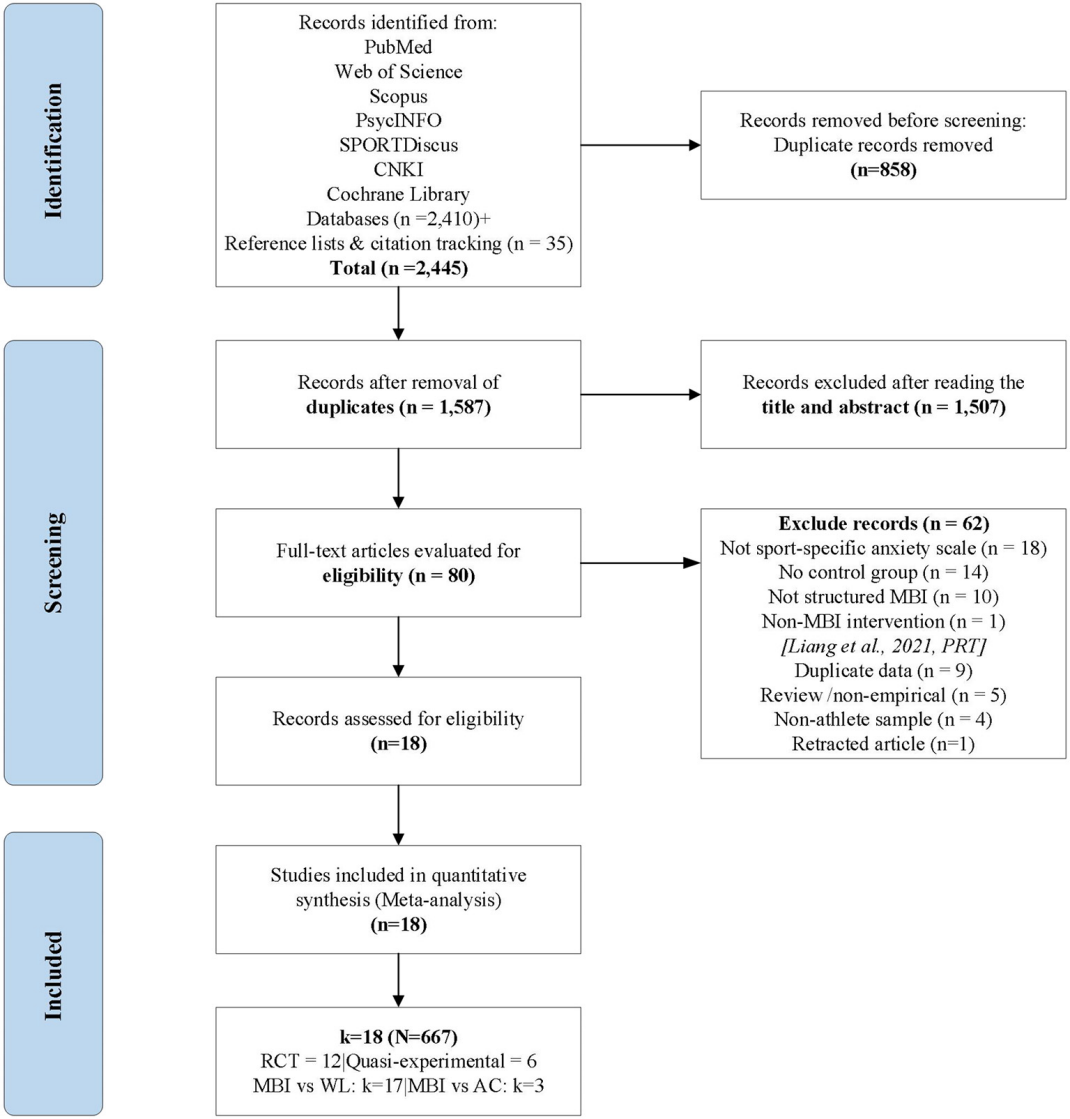
PRISMA flow diagram of study selection. Adapted from the PRISMA 2020 statement (Page et al., 2021).

The literature screening process is shown in Figure 1. An initial search yielded approximately 2,410 records from seven databases, and a supplementary search (reference tracing and gray literature) yielded 35 more, for a total of 2,445 records. After removing 858 duplicate records, 1,587 records were retained for title and abstract screening. At this stage, 1,507 records that clearly did not meet the inclusion criteria were excluded, mainly because they were non-intervention studies, non-athlete samples, or unrelated to the mindfulness/anxiety topic. Subsequently, eligibility was assessed on 80 full-text articles, and 62 were excluded. Specific reasons for exclusion were: using only general anxiety scales instead of sports-specific competition anxiety scales (*n* = 18); single-group pre- and post-test designs without a control group (*n* = 14); interventions with less than 50% mindfulness component or unstructured protocols (*n* = 10); interventions themselves being non-mindful methods (*n* = 1, i.e., progressive muscle relaxation training in Liang et al., 2021); using the same dataset as included studies or containing unverifiable duplicate data (*n* = 9); reviews, commentaries, or non-empirical studies (*n* = 5); participants being non-athletes in the general population (*n* = 4); and retracted literature (*n* = 1, i.e., Ning et al., 2022). Ultimately, 18 studies were included in the quantitative synthesis.

### 3.2 Inclusion characteristics

As shown in Table 1, the 18 studies involved a total of 667 participants (344 in the intervention group and 323 in the control group). The sample size of each study ranged from 16 (Hoja and Jansen, 2019) to 160 (Naderi et al., 2020), with a median of 35. The studies were published between 2016 and 2025, from 10 countries. China (*n* = 4) and Iran (*n* = 4) accounted for 44% of the total, the United States (*n* = 2) and Hungary (*n* = 2) each contributed two studies, and Australia, Indonesia, Peru, Germany, Spain, and Sweden each contributed one study. Applying the Hofstede individualism–collectivism cut-off to the country of data collection, ten studies were conducted in collectivist cultures (China, Iran, Indonesia, and Peru) and eight studies in individualist cultures (the United States, Australia, Hungary, Germany, Spain, and Sweden).

**Table 1:** Characteristics of included studies (*k* = 18).

Study	*N* (MBI/C)	Design	Gender	Age	Country	Sport	Level	MBI	Duration	Control	Measure	*g*
Scott-Hamilton et al. (2016)	47 (27/20)	RCT	Mixed	39.8	Australia	Cycling	University	MBSR-ad	8 week/8 s/30 min	WL	SAS-2	−0.85
Kusuma and Bin (2017)	20 (10/10)	QE	Mixed	—	Indonesia	Badminton	Local	MBSR-ad	6 week/18 s/60 min	NI	CSAI-2	−0.68
(Trujillo-Torrealva and Reyes Bossio (2019)	33 (16/17)	RCT	Mixed	18.9	Peru	Martial arts	University	Other	6 week/12 s/60 min	NI	CSAI-2	−0.73
Dehghani et al. (2018)	31 (15/16)	RCT	Male	24.0	Iran	Basketball	University	MAC	8 week/8 s/90 min	NI	SCAT	−0.61
Hoja and Jansen (2019)	16 (8/8)	QE	Mixed	26.5	Germany	Tennis	National	MBSR-ad	7 week/7 s/120 min	NI	CAI-T	−0.66
Mehrsafar et al. (2019)	26 (13/13)	RCT	Male	24.3	Iran	Wushu	National	MBSR-ad	8 week/8 s/60 min	WL	CSAI-2	−0.61
Danran et al. (2020)	49 (25/24)	RCT	Mixed	19.5	China	Badminton	National	MAIC	7 week/7 s/90 min	NI	SCAT	−0.62
Naderi et al. (2020)	160 (81/79)	RCT	Male	17.1	Iran	Soccer	Local	MAC	7 week/7 s/45 min	Lecture	SAS-2	−0.75
Wolch et al. (2021)	32 (16/16)	RCT	Male	21.2	USA	Basketball	University	MBSR-ad	6 week/6 s/15 min	NI	CSAI-2	−0.64
Dana et al. (2022) ^a^	45 (15/15/15)	QE	Male	24.5	Iran	Shooting	Local	MSPE	6 week/6 s/60 min	NI+PST	CSAI-2	−0.66/−0.11
Gan et al. (2024)	50 (23/27)	RCT	Mixed	21.9	China	Soccer/Tennis	National	MAIC	8 week/8 s/75 min	NI	CSAI-2	−0.59
Sánchez-Sánchez et al. (2023)	41 (21/20)	RCT	Mixed	22.8	Spain	Soccer/Tennis	National	MAC	10 week/10 s/40 min	WL	CSAI-2R	−0.66
Wang et al. (2023a)	43 (23/20)	QE	Male	20.9	China	Basketball	University	MAIC	7 week/7 s/60 min	NI	SCAT	−0.31
Hut et al. (2023) ^‡^	24 (15/9)	RCT	Mixed	20.1	USA	Track/Field	University	MSPE	6 week/12 s/45 min	PST	SAS-2	0.08
Tóth et al. (2023) ^a, ‡^	42 (14/14/14)	QE	Mixed	21.4	Hungary	Mixed	University	Other	6 week/6 s/60 min	NI+REBT	CSAI-2R	−0.64/−0.15
Yu et al. (2024)	34 (17/17)	RCT	Mixed	20.8	China	Table tennis	National	MAIC	7 week/7 s/90 min	NI	CSAI-2	−0.53
Josefsson et al. (2024)	30 (15/15)	RCT	Male	23.6	Sweden	Martial arts	National	MAC	8 week/8 s/60 min	NI	SAS-2	−0.62
Kelemen et al. (2025)	20 (10/10)	QE	Mixed	21.5	Hungary	Distance runners	National	MSPE	6 week/6 s	NI	CSAI-2	−0.62

RCT, randomized controlled trial; QE, quasi-experimental; MBSR-ad, adapted MBSR; MAC, mindfulness-acceptance-commitment; MAIC, mindfulness-acceptance-insight-commitment; MSPE, mindful sport performance enhancement; WL, waitlist; NI, no intervention; PST, psychological skills training; REBT, rational emotive behavior therapy; SAS-2, Sport Anxiety Scale-2; CSAI-2, Competitive State Anxiety Inventory-2; CSAI-2R, CSAI-2 Revised; SCAT, Sport Competition Anxiety Test; CAI-T, Competition Anxiety Inventory-Trait. Dash indicates not reported.

^a^Three-arm study. Two *g* values reported: MBI vs. waitlist / MBI vs. active control. MBI group sample halved per comparison.

^‡^Active control comparison study.

The average age of participants ranged from 17.1 years (Naderi et al., 2020) to 39.8 years (Scott-Hamilton et al., 2016), with an overall weighted average age of approximately 22.4 years. Regarding gender, eight studies had exclusively male samples, eight had mixed-sex samples, two did not explicitly report the gender ratio, and there were no studies with exclusively female samples. The sport categories represented in the primary samples were football (*k* = 1), basketball (*k* = 3), badminton (*k* = 2), shooting (*k* = 1), tennis (*k* = 1), martial arts including wushu (*k* = 3), track and field (*k* = 1), cycling (*k* = 1), table tennis (*k* = 1), long-distance running (*k* = 1), and mixed sports (*k* = 3). In terms of athletic level, there were six national/regional athletes, seven university athletes, and five local/amateur athletes.

Regarding study design, there were 12 RCTs and six quasi-experimental designs. The MBI type distribution is as follows: five MBSR or MBSR-adapted versions (Scott-Hamilton et al., 2016; Kusuma and Bin, 2017; Hoja and Jansen, 2019; Mehrsafar et al., 2019; Wolch et al., 2021), four MAC (Dehghani et al., 2018; Naderi et al., 2020; Sánchez-Sánchez et al., 2023; Josefsson et al., 2024), four MAIC (Danran et al., 2020; Gan et al., 2024; Wang et al., 2023a; Yu et al., 2024), and three MSPE (Dana et al., 2022; Hut et al., 2023; Kelemen et al., 2025), and two other types (Trujillo-Torrealva and Reyes Bossio, 2019; Tóth et al., 2023). The duration of interventions ranged from 6 to 10 weeks (Mdn = 7 weeks), the duration of each session ranged from 15 to 120 min, and the total intervention duration ranged from 6 to 16 h (Mdn = 10.5 h).

Regarding control types, 15 studies included only a wait/no treatment control, two were three-arm designs (including MBI, active control, and wait control; Dana et al., 2022; Tóth et al., 2023), and one included only an active control (Hut et al., 2023). Therefore, the extractable comparison *k* = 17 for MBI vs. wait control and the extractable comparison *k* = 3 for MBI vs. active control. The three active controls were psychological skills training (PST), psychological skills program, and rational emotive behavior therapy (REBT). The control group in Naderi et al. (2020) received routine physical education lectures, the content of which was unrelated to anxiety management and did not include any structured mental skills training components; therefore, they were classified as attention controls rather than active intervention controls. The competition anxiety scales were distributed as follows: CSAI-2 or CSAI-2R (*k* = 10), SAS-2 (*k* = 4), SCAT (*k* = 3), and CAI-T (*k* = 1).

### 3.3 Risk of bias

**Figure 2:**
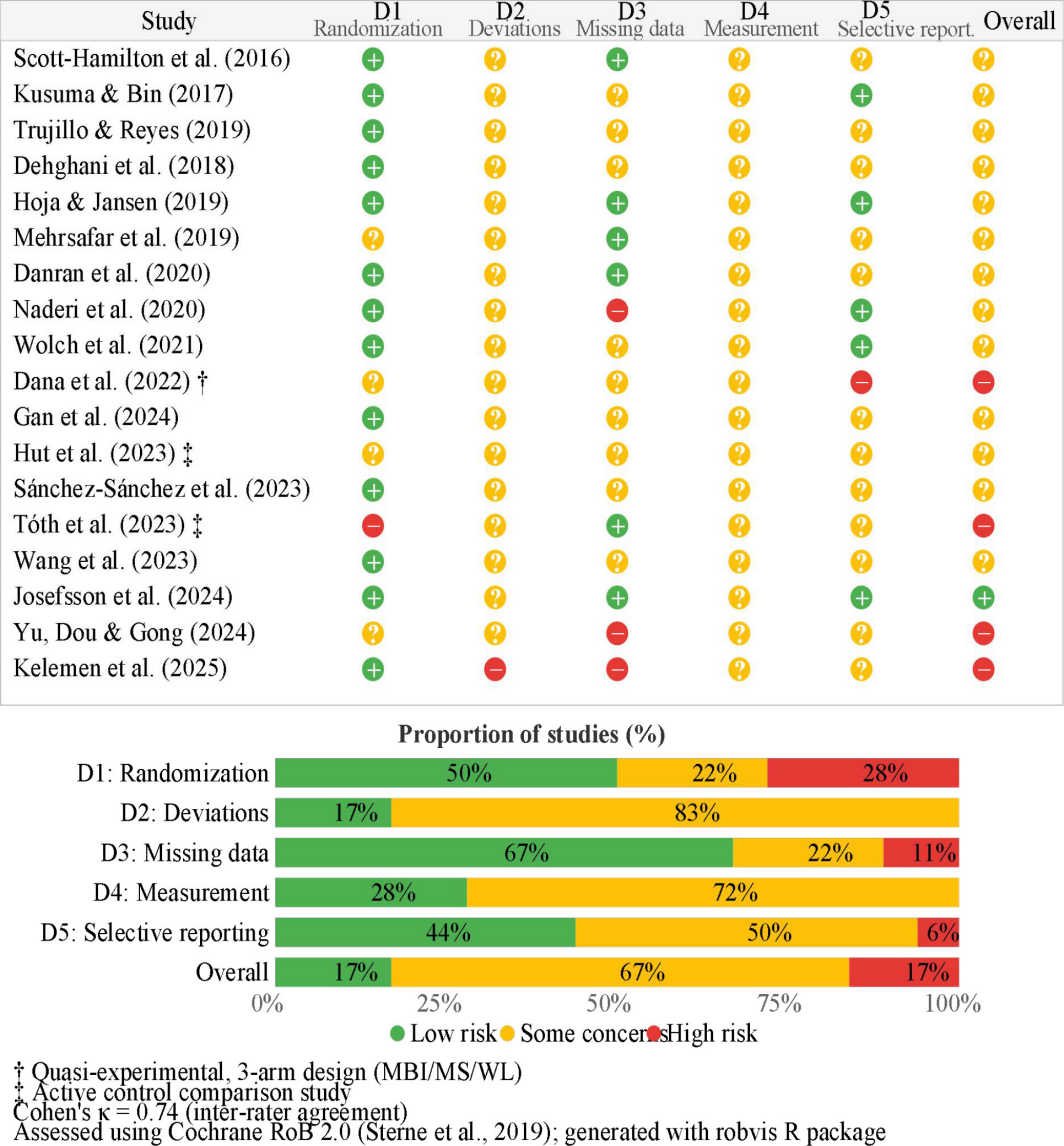
Risk of bias summary across included studies. Assessed using the cochrane risk of bias 2.0 tool. D1, randomization process; D2, deviations from intended interventions; D3, missing outcome data; D4, measurement of the outcome; D5, selection of the reported result.

As shown in Figure 2, the agreement between the two raters was good (Cohen's Kappa = 0.74). In the D1 randomization process domain, nine items (50%) were low risk, four items (22%) were partially concerning, and five items (28%) were high risk—the latter being quasi-experimental designs that did not employ randomization. Tóth et al. (2023) claimed randomization but assigned incapacitated participants to the control group; their D1 was also rated as high risk. In the D2 deviation from the intended intervention domain, three items (17%) were low risk, 15 items (83%) were partially concerning, and no items were high risk—this distribution reflects the inherent limitation of mindfulness interventions that cannot blind participants. In the D3 missing outcome data domain, 12 items (67%) were low risk, four items (22%) were partially concerning, and two items (11%) were high risk. In the D4 outcome measurement domain, five items (28%) were low risk and 13 items (72%) were partially concerning, reflecting the inherent limitations of the self-report scale in its open-label design. In the D5 selective reporting domain, eight items (44%) were low risk, nine items (50%) were partially concerning, and one item (6%) was high risk. Overall risk of bias: three items (17%) were low risk, 12 items (67%) were partially concerning, and three items (17%) were high risk.

### 3.4 Overall effect

**Figure 3:**
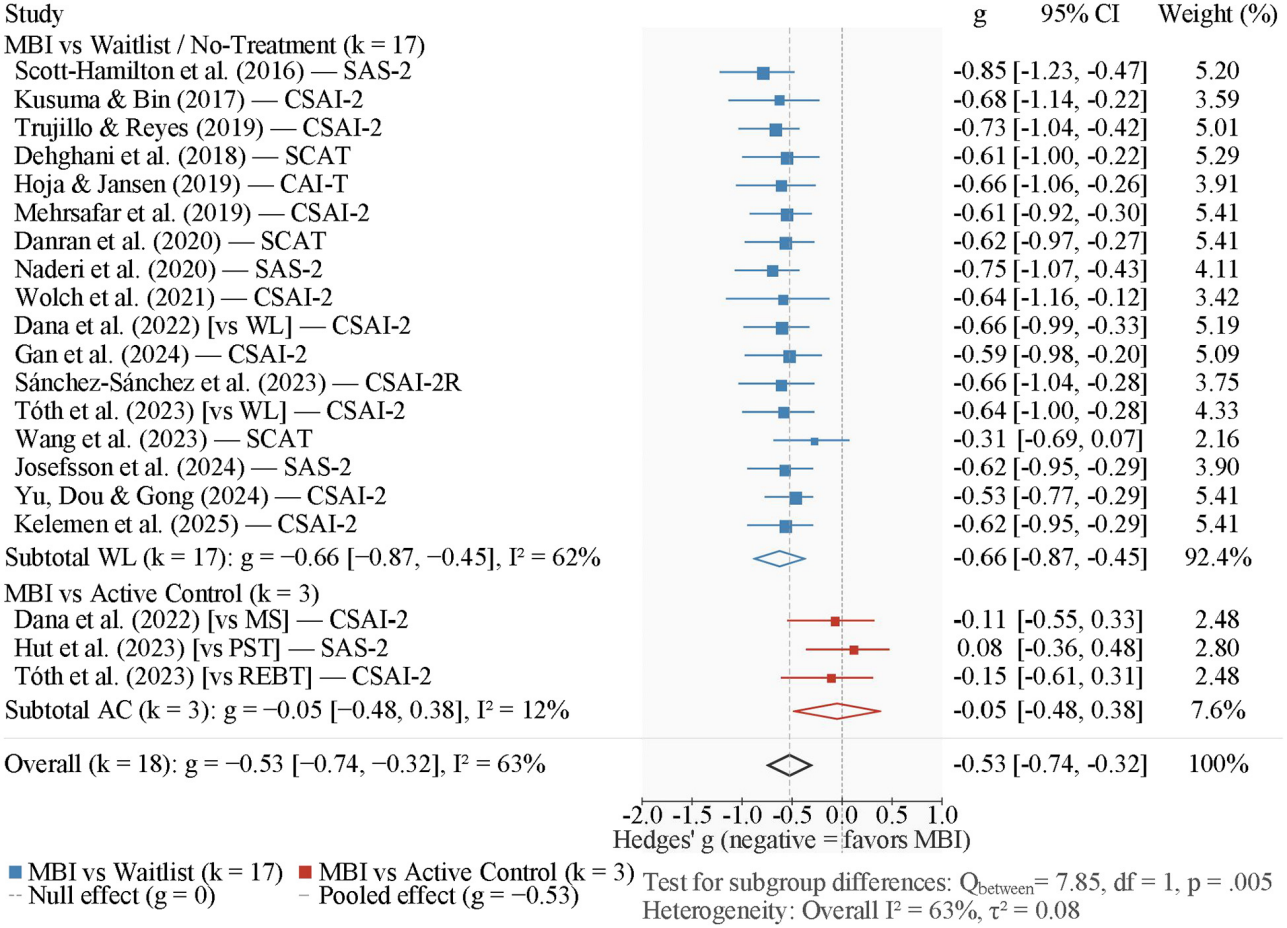
Forest plot of MBI effects on competitive anxiety. Effect sizes are Hedges' *g* with 95% CIs. Negative values indicate lower anxiety in the MBI group. The diamond represents the pooled estimate. Studies are ordered by publication year within each subgroup. Subgroup subtotals are based on 20 comparisons (three-arm studies split by control type with MBI sample halved); the overall diamond is based on 18 study-level effect sizes.

As shown in Figure 3, of the 20 comparisons, 19 had negative effect sizes (i.e., lower anxiety in the MBI group than in the control group), with only the active control comparison in Hut et al. (2023) showing a positive effect size (*g* = 0.08). Individual effect sizes ranged from *g* = −0.85 (Scott-Hamilton et al., 2016) to *g* = 0.08 (Hut et al., 2023 vs PST). In the overall analysis, the three-arm studies (Dana et al., 2022; Tóth et al., 2023) pooled the effect sizes of the two control groups at the study level to avoid duplicate counting of the MBI group sample; therefore, the overall *k* = 18. In the subgroup analyses by control type, the three-arm studies were split into independent comparisons (the MBI group sample was halved), resulting in *k* = 20 comparisons. The pooled effect size was moderate [*k* = 18, *g* = −0.53, 95% CI (−0.74, −0.32), *p* < 0.001], with moderately high heterogeneity [*Q* = 45.86, df = 17, *p* < 0.001; *I*^2^ = 63%, 95% CI (38%, 77%)]. Dimension-specific analyses drew on the subset of studies using multidimensional instruments (CSAI-2/CSAI-2R and SAS-2). The cognitive-anxiety and somatic-anxiety estimates pool the subcomponents shared by the two instruments, whereas the self-confidence estimate is restricted to CSAI-2/CSAI-2R. Cognitive anxiety showed the largest benefit [*k* = 13, *g* = −0.58, 95% CI (−0.82, −0.34), *p* < 0.001, *I*^2^ = 67%], with effect sizes ranging from *g* = −1.34 to *g* = 0.08 in individual studies. Somatic anxiety had the second largest effect size [*k* = 13, *g* = −0.41, 95% CI (−0.64, −0.18), *p* < 0.001, *I*^2^ = 59%], ranging from *g* = −1.12 to *g* = 0.15. Self-confidence was extracted only from the CSAI-2/CSAI-2R subset [*k* = 9, *g* = +0.35, 95% CI (0.13, 0.57), *p* = 0.002, *I*^2^ = 55%], indicating that the MBI group had higher self-confidence than the control group, ranging from *g* = −0.10 to *g* = 0.82. The effect sizes of all three dimensions reached statistical significance, but the level of heterogeneity suggests the existence of moderating factors. Dana et al. (2022) administered the CSAI-2 but reported only the total score; the study therefore contributes to the overall synthesis but not to the dimension-specific analyses.

### 3.5 Analysis of moderating variables

As shown in Table 2, the control type was the only statistically significant category moderating variable (*Q*_between = 7.85, *p* = 0.005). The effect of MBI compared to the waitlist control was moderate [*k* = 17, *g* = −0.66, 95% CI (−0.87, −0.45), *p* < 0.001, *I*^2^ = 62%], with 15 effect sizes negative and two approaching zero within this subgroup. The effect of the MBI compared to the active control was close to zero [*k* = 3, *g* = −0.05, 95% CI (−0.48, 0.38), *p* = 0.82, *I*^2^ = 12%]. The effect sizes of the three studies were *g* = 0.08 (Hut et al., 2023), *g* = −0.11 (Dana et al., 2022), and *g* = −0.15 (Tóth et al., 2023), respectively, showing a directionally consistent pattern and none reaching a significant level.

**Table 2:** Categorical moderator analyses.

Moderator/level	*k*	*g*	95% CI	*p*	*I^2^* (%)	*Q*_between	*p*_between
Control type							
Waitlist/NI	17	−0.66	(−0.87, −0.45)	<0.001	62	7.85	0.005
Active control	3	−0.05	(−0.48, 0.38)	0.82	12		
MBI type							
MAIC	4	−0.54	(−0.86, −0.22)	0.001	46	1.32	0.724
MAC	4	−0.55	(−0.88, −0.22)	0.001	42		
MBSR-adapted	5	−0.46	(−0.91, −0.01)	0.044	72		
MSPE	3	−0.30	(−0.74, 0.14)	0.18	18		
Other	2	—	—	—	—		
Sport type							
Individual	9	−0.54	(−0.80, −0.28)	<0.001	53	0.44	0.508
Team	6	−0.48	(−0.86, −0.10)	0.013	71		
Mixed	3	−0.58	(−1.04, −0.12)	0.014	44		
Competitive level							
National/Regional	6	−0.62	(−0.98, −0.26)	<0.001	73	0.96	0.327
University/Local	12	−0.47	(−0.70, −0.24)	<0.001	56		
Cultural background							
Collectivist	10	−0.58	(−0.84, −0.32)	<0.001	63	0.85	0.356
Individualist	8	−0.45	(−0.75, −0.15)	0.003	68		
Scale type							
State (CSAI-2/2R)	9	−0.58	(−0.86, −0.30)	<0.001	71	0.28	0.597
Trait (SAS-2/SCAT/CAI-T)	9	−0.46	(−0.74, −0.18)	0.001	58		

*k*, number of comparisons; *g*, Hedges' *g* (negative = lower anxiety in MBI group); *Q*_between = between-group heterogeneity. Active control includes PST and REBT. Collectivist cultures = China, Iran, Indonesia, Peru; Individualist cultures = United States, Australia, Hungary, Germany, Spain, Sweden. Analyses conducted when *k* ≥ 3.

Regarding the type of MBI, MAIC had the highest effect size [*k* = 4, *g* = −0.54, 95% CI (−0.86, −0.22), *I*^2^ = 46%], followed by MAC [*k* = 4, *g* = −0.55, 95% CI (−0.88, −0.22), *I*^2^ = 42%], MBSR was in the middle [*k* = 5, *g* = −0.46, 95% CI (−0.91, −0.01), *I*^2^ = 72%], and MSPE had the lowest [*k* = 3, *g* = −0.30, 95% CI (−0.74, 0.14), *I*^2^ = 18%]. There were no significant differences between groups (*Q*_between = 1.32, *p* = 0.724). It is noteworthy that two out of the three studies in the MSPE subgroup used active controls.

Regarding the type of sport, the effect sizes were similar for individual events [*k* = 9, *g* = −0.54, 95% CI (−0.80, −0.28), *I*^2^ = 53%] and team events [*k* = 6, *g* = −0.48, 95% CI (−0.86, −0.10), *I*^2^ = 71%]. The effect size was slightly higher in the mixed events subgroup [*k* = 3, *g* = −0.58, 95% CI (−1.04, −0.12), *I*^2^ = 44%], but the difference between groups was not significant (*Q*_between = 0.44, *p* = 0.508). Regarding athletic ability, the effect size of elite athletes [*k* = 6, *g* = −0.62, 95% CI (−0.98, −0.26), *I*^2^ = 73%] was slightly higher than that of college/amateur athletes [*k* = 12, *g* = −0.47, 95% CI (−0.70, −0.24), *I*^2^ = 56%], but the difference was not significant (*Q*_between = 0.96, *p* = 0.327). With respect to cultural background operationalized through Hofstede's individualism–collectivism dimension, the pooled estimate in the collectivist-culture stratum [*k* = 10, *g* = −0.58, 95% CI (−0.84, −0.32), *I*^2^ = 63%] was numerically larger in magnitude than the estimate in the individualist-culture stratum [*k* = 8, *g* = −0.45, 95% CI (−0.75, −0.15), *I*^2^ = 68%], but the between-stratum comparison did not reach significance (*Q*_between = 0.85, *p* = 0.356). Collectivist cultures were China, Iran, Indonesia, and Peru; individualist cultures were the United States, Australia, Hungary, Germany, Spain, and Sweden. Regarding scale type, there was no significant difference between the state scale [*k* = 9, *g* = −0.58, 95% CI (−0.86, −0.30), *I*^2^ = 71%] and the trait scale [*k* = 9, *g* = −0.46, 95% CI (−0.74, −0.18), *I*^2^ = 58%] (*Q*_between = 0.28, *p* = 0.597).

### 3.6 Meta-regression

**Figure 4:**
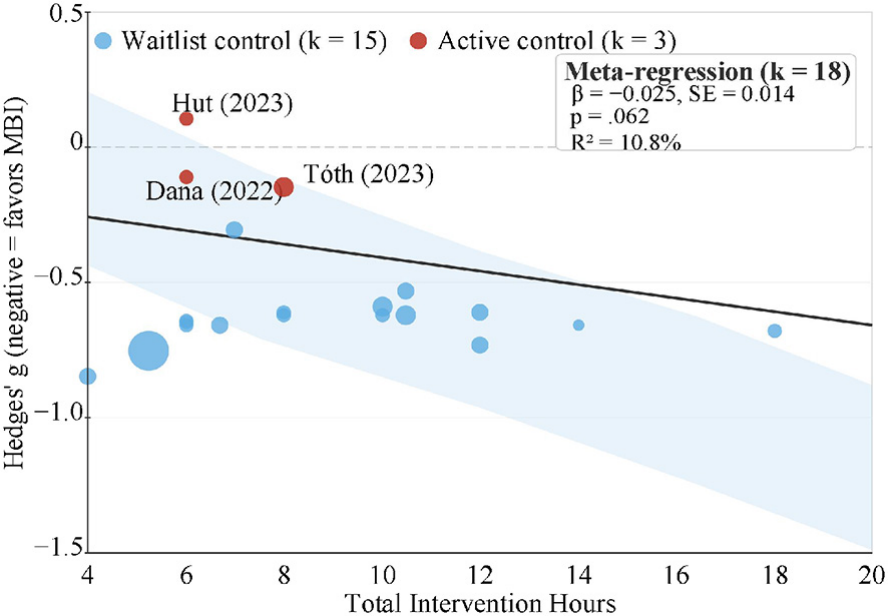
Meta-regression of total intervention hours on effect size. Each circle represents one study; circle size is proportional to study weight. Red circles indicate active control comparisons. The solid line represents the regression slope (β = −0.025, *p* = 0.062).

As shown in Table 3 and Figure 4, in the univariate meta-regression, the coefficient for total intervention duration (β = −0.025, SE = 0.014, *p* = 0.062, *R*^2^ = 10.8%) did not reach the conventional significance threshold. The direction of the coefficient, whereby each additional hour of intervention was associated with an approximately 0.025-standard-deviation-unit decrease in the effect estimate, is reported here for descriptive completeness. The coefficient for study sample size likewise did not reach significance (β = 0.006, SE = 0.003, *p* = 0.056, *R*^2^ = 8.2%). Larger studies tended to report smaller absolute effects, and this pattern is interpreted together with the funnel-plot findings below rather than as evidence for an independent sample-size effect. The number of weeks of intervention (β = −0.033, SE = 0.036, *p* = 0.348), year of publication (β = −0.026, SE = 0.021, *p* = 0.198), and mean age (β = −0.004, SE = 0.012, *p* = 0.698) were all not significant. A multivariate model that included both total intervention duration and sample size explained 16.2% of the heterogeneity (QM = 7.48, df = 2, *p* = 0.024). As shown in Figure 4, active control studies (marked in red) were concentrated in the shorter intervention duration range (6–8 h), while the intervention duration of waitlist control studies was more widely distributed (6–18 h).

**Table 3:** Meta-regression results.

Predictor	β	SE	*p*	*R*^2^ (%)
Univariate models				
Total intervention hours	−0.025	0.014	0.062	10.8
Sample size	0.006	0.003	0.056	8.2
Intervention weeks	−0.033	0.036	0.348	1.8
Publication year	−0.026	0.021	0.198	3.8
Mean age	−0.004	0.012	0.698	0.0
Multivariate model				16.2
Total intervention hours	−0.023	0.013	0.068	—
Sample size	0.005	0.003	0.074	—

β, unstandardized coefficient; *R*^2^, proportion of heterogeneity explained. Multivariate model: QM = 7.48, df = 2, *p* = 0.024. Negative β for hours indicates more intervention time associated with greater anxiety reduction.

### 3.7 Publication bias

**Figure 5:**
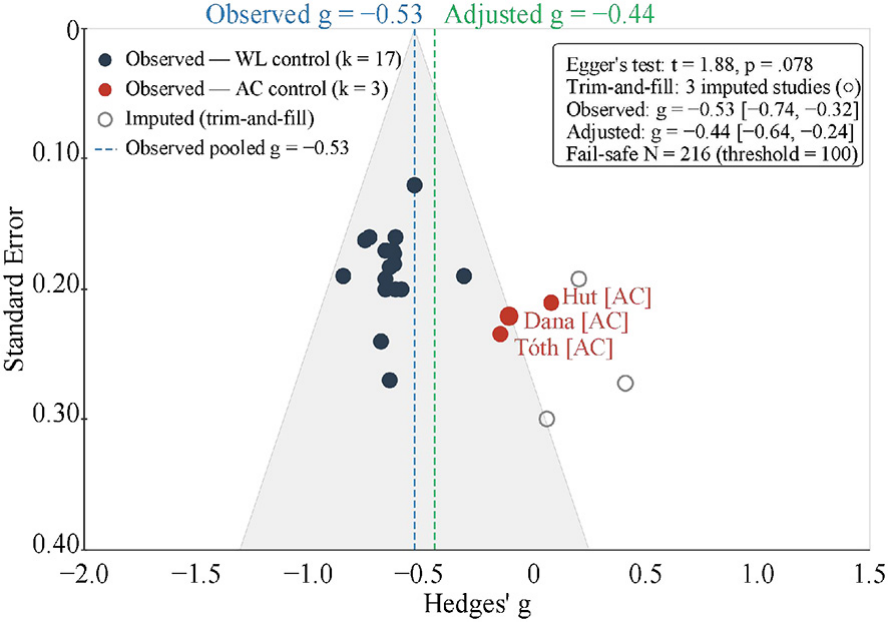
Funnel plot with trim-and-fill estimate*s*. Open circles represent observed studies; filled circles represent imputed studies. The vertical dashed line indicates the adjusted pooled estimate (*g* = −0.44).

As shown in Figure 5, the funnel plot exhibits mild right-side asymmetry, indicating a tendency for negative effect sizes to be larger in small-sample studies. The Egger regression test did not reach significance (*t* = 1.88, df = 16, *p* = 0.078), providing insufficient evidence of publication bias, but the direction suggests the possible existence of a mild small-study effect. Duval and Tweedie's trim-and-fill method estimated three missing studies on the right side, and the adjusted effect size was changed from *g* = −0.53 to *g* = −0.44 [95% CI (−0.64, −0.24)], still maintaining statistical significance. The Rosenthal Fail-safe *N* was 216, far exceeding the threshold of 5k + 10 = 100, indicating that even with the existence of unpublished zero-effect studies, the overall conclusions remain robust. Based on the four indicators, publication bias poses a limited threat to the conclusions of this meta-analysis, but the effect size after trim-and-fill correction (*g* = −0.44) may be closer to the true effect.

### 3.8 Sensitivity analysis

The elimination method showed that the pooled effect size ranged from *g* = −0.49 to *g* = −0.57 after each study was removed sequentially, all maintaining statistical significance. No single study had a decisive impact on the overall conclusion. The effect size increased slightly after removing Naderi et al. (2020) (*g* = −0.49), while the effect size changed the least after removing Scott-Hamilton et al. (2016) (*g* = −0.50), but the changes were within a reasonable range.

After removing three studies with a high risk of bias and re-pooling (*k* = 15), *g* = −0.48 [95% CI (−0.69, −0.27), *I*^2^ = 55%]. Heterogeneity decreased, but the direction and significance of the conclusions remained unchanged. After re-including the five studies from Wang et al. (2024) that used only the general anxiety scale (*k* = 23), the pooled effect size was *g* = −0.51 [95% CI (−0.68, −0.34)], which was very close to the original estimate. The analysis of studies using only the state scale (*k* = 9) had a pooled effect size of *g* = −0.58 [95% CI (−0.86, −0.30), *I*^2^ = 71%], slightly larger than the overall effect size. The analysis of RCTs (*k* = 12) had a pooled effect size of *g* = −0.55 [95% CI (−0.79, −0.31), *I*^2^ = 59%], largely consistent with the overall result. Furthermore, after reclassifying Naderi et al. (2020) as an active control, the pooled effect size for the active-control subgroup (*k* = 4) was *g* = −0.25 [95% CI (−0.57, 0.07)], with no change to the overall conclusions. A complementary sensitivity analysis that used only the waitlist-arm comparison (rather than the weighted-mean combination of both control arms) for each three-arm study yielded an overall pooled estimate of *g* = −0.55 [95% CI (−0.76, −0.34), *k* = 18], essentially unchanged from the primary estimate and indicating that the overall synthesis is robust to the handling of the three-arm studies. The results of the above sensitivity analyses are consistent, indicating that the overall conclusions of this meta-analysis are robust to changes in inclusion criteria, quality screening, and study design.

## 4 Discussion

This meta-analysis included 18 controlled trials to examine the effect of MBI on athletes' competitive anxiety. It found that MBI produced a moderate anxiety-relief effect (*g* = −0.53), with cognitive anxiety benefiting more than physical anxiety benefit, while the improvement in self-confidence was relatively limited. Control type was the only statistically significant categorical moderator; the MBI effect was almost entirely driven by the waitlist-control comparison, while the effect compared to the active control was close to zero. A non-significant numerical tendency was observed between total intervention duration and effect size. The following discussion focuses on these core findings.

### 4.1 The anxiety-relieving effect of mindfulness interventions and its dimensional differences

The overall effect size (*g* = −0.53) was smaller than the *g* = −0.67 reported by Wang et al. (2024). The difference from the pooled estimate reported by Wang et al. (2024) is likely attributable to two classes of factor acting jointly. Methodological refinements contributed substantively, including strict limitation to sport-specific competition-anxiety instruments, exclusion of the general anxiety instruments used in the earlier synthesis (BAI in Norouzi et al., 2020; STAI in Solberg et al., 2000; DASS in Goodman et al., 2014), removal of the progressive-muscle-relaxation comparison in Liang et al. (2021), correction of the coding in Dana et al. (2022), and exclusion of the retracted Ning et al. (2022). Sample-composition shifts are also plausible contributors, because the present synthesis incorporated three active-control comparisons with near-zero effects and drew from a more recent cohort of studies in which comparator design has gradually shifted toward more stringent conditions (Cuijpers et al., 2024; Michopoulos et al., 2021). The relative contributions of these two classes cannot be precisely partitioned with the available data and are flagged as a limitation. The effect size of this study is largely consistent with the SMD (−0.55) of the mindfulness subgroup in Li et al. (2025), and also with the findings of Zhang et al. (2025) and Reinebo et al. (2024), enhancing the convergent validity of the results.

Dimensional analysis revealed that the benefit in cognitive anxiety (*g* = −0.58) was greater than that in somatic anxiety (*g* = −0.41), a pattern compatible with the core mechanisms of mindfulness proposed in the literature. Mindfulness reduces rumination on threatening thoughts through cognitive dissociation (Birrer et al., 2012), resulting in a more direct improvement at the cognitive level, consistent with the systematic review by Goldberg et al. (2022) and the meta-analysis on mindfulness and flow connectivity by Schutte and Malouff (2023). While the positive improvement in self-confidence (*g* = 0.35) was significant, its magnitude was small, possibly reflecting that mindfulness exerts its influence through indirect emotion regulation pathways rather than direct self-confidence building mechanisms (Wang et al., 2023b). This finding suggests that if the primary goal is to boost self-confidence, it may be necessary to integrate targeted strategies such as goal setting and positive self-talk into MBI (Gardner and Moore, 2004).

### 4.2 The issue of non-specific effects revealed by control type

Control type was the only statistically significant categorical moderator (*Q*_between = 7.85, *p* = 0.005), and the magnitude of the between-stratum contrast was larger than commonly observed for moderator effects in adjacent literatures. Relative to waitlist comparators, MBI were associated with a moderate reduction in competitive anxiety (*g* = −0.66). Relative to active comparators, the pooled estimate approached zero (*g* = −0.05, 95% CI [−0.48, 0.38]). The latter estimate rests on only three comparisons and carries a correspondingly wide confidence interval, so the numerical near-identity with zero should not be read as evidence for equivalence. The data are compatible with a range of small-to-moderate true differences in either direction between MBI and established active alternatives. This pattern emerges from three independent studies. Hut et al. (2023) found in an RCT of MSPE vs. PST that the two groups of athletes showed comparable improvements in athletic anxiety (*g* = 0.08), with MSPE showing an advantage only in athletic performance satisfaction (Hut et al., 2021; Minkler et al., 2021). The three-arm design of Dana et al. (2022) also showed no significant difference in the reduction of athletic anxiety between mindfulness and mental skills training (*g* = −0.11). In Tóth et al. (2023), REBT significantly improved both cognitive and somatic anxiety, and the mindfulness group likewise showed a modest, non-significant reduction, with a small between-group difference slightly favoring MBI (*g* = −0.15).

This pattern suggests that the anxiety-reducing effect of MBI observed in previous meta-analyses (Wang et al., 2024, *g* = −0.67) may primarily reflect non-specific effects—including treatment expectations, social interaction, attention effects, and experimenter effects—rather than the unique contribution of mindfulness-specific mechanisms. This pattern is compatible with the phenomenon of effect size inflation caused by waitlist controls observed by Cuijpers et al. (2024) in the field of clinical depression, and also echoes the study by Michopoulos et al. (2021) on different effect estimates produced by different control conditions. Within sports psychology, Ong and Chua (2021) reported an effect size of *g* = −0.42 for traditional mental skills training on competitive anxiety, while Myall et al. (2023) 's meta-analysis of MBI on the mental health of elite athletes also suggested a similar non-specific effect pattern. RCTs in the sports field also support this interpretation: Röthlin et al. (2020) found differentiated but shared effects after comparing mental skills training and mindfulness training, and Lundgren et al. (2020, 2021)'s ACT study in ice hockey players also did not find a unique anxiety-reducing effect beyond the control group.

A cautionary note concerns the active-control stratum, which comprises only three studies and yields a correspondingly wide confidence interval [95% CI (−0.48, 0.38)]. The three studies originate from three different countries (United States, Iran, Hungary), employ different mindfulness protocols (MSPE, MSPE, MMTS 2.0), and pair mindfulness-based intervention with different active comparators (psychological skills training, mental skills training, and rational-emotive behavior therapy), and the three point estimates converge in the neighborhood of zero. Descriptive consistency across heterogeneous contexts strengthens the plausibility of the observed pattern, but such qualitative convergence does not substitute for the statistical power that only additional well-powered trials can provide. The near-null active-control estimate should accordingly be regarded as preliminary and as a hypothesis for replication rather than as a settled finding, and adequately powered head-to-head randomized trials remain a priority for the field.

### 4.3 Intervention dosage and small sample effect

In the meta-regression, the coefficient for total intervention duration (β = −0.025, *p* = 0.062, *R*^2^ = 10.8%) did not reach the conventional significance threshold and should be interpreted as a non-significant numerical tendency rather than as evidence for a dose-response effect. Furthermore, this numerical tendency may itself be confounded with control type: as shown in Figure 4, the intervention duration in active-control studies (6–8 h) was generally shorter than the median in waitlist-control studies (10.5 h), so the apparent association between total duration and effect size may partially reflect the influence of control type rather than a pure dose effect. The coefficient for sample size (β = 0.006, *p* = 0.056) did not reach significance, and the direction was consistent with the Egger test results, suggesting a possible small-study effect in this literature. This finding is consistent with observations by Singh et al. (2023) and Ramos-Sanchez et al. (2021) in adjacent areas. The median sample size in the included studies was only 35 participants, and most studies (*k* = 12) had sample sizes of fewer than 50 participants. This generally insufficient statistical power suggests that future research urgently needs to make more thorough preparations in sample planning.

It is noteworthy that most effect sizes in the waitlist-control subgroup were concentrated in the range of *g* = −0.59 to −0.68, despite significant differences in sample size, scales, and cultural background among the included studies. This pattern may reflect the relative stability of non-specific effects in waitlist-control designs, but it also does not rule out the possibility of convergent outcome reporting in this field.

### 4.4 Practical implications

MBI can be considered one option among several for supporting the management of competitive anxiety in athletic populations, with the available evidence pointing most clearly to benefits in the cognitive-anxiety component. The meta-regression coefficient for total intervention duration did not reach significance, so specific dose prescriptions are not warranted on the basis of the present data. The numerical tendency is compatible with protocols lasting 6 to 8 weeks and accumulating approximately ten or more contact hours, and this configuration may be treated as a reasonable default pending dose-titration trials. The MAC and MAIC programmes carry the broadest evidence base among the intervention families examined. Practitioners should nonetheless note that the present evidence does not establish MBI as superior to established active alternatives such as psychological skills training or rational-emotive behavior therapy for anxiety reduction, while acknowledging that this inference rests on only three head-to-head comparisons. The choice of a mindfulness programme in applied settings is therefore better anchored in individual athlete preferences and in secondary targets such as cultivated acceptance and attentional flexibility than in claims of superiority for anxiety outcomes. The selection of MBI should be based on the athlete's personal preferences and specific goals (such as improving mindfulness and acceptance), rather than on assumptions of superiority in anxiety management. Athlete mental health support should adopt a diversified strategy (Henriksen et al., 2020; Schinke et al., 2024; Åkesdotter et al., 2020; Purcell et al., 2020; Gouttebarge et al., 2021; Mountjoy et al., 2023), incorporating mindfulness as one option in the toolkit.

### 4.5 Limitations and future directions

This study has several limitations. Mindfulness interventions cannot be *blinded*, and all studies have an inherent risk of bias in the D2 domain, a methodological challenge that is difficult to avoid in this field. The measurement of competition anxiety relies on self-report scales and does not include objective physiological indicators such as heart rate variability or cortisol, which may lead to social expectation bias. Several features of the evidence base constrain the certainty of our inferences. The active-control stratum contained only three comparisons. Given the observed within-stratum sampling variance (implied pooled SE ≈ 0.219 from the stratum 95% CI), a formal power calculation indicates that this stratum has approximately 80% power to detect pooled differences of roughly |*g*| ≥ 0.61 between MBI and active comparators. Small true advantages of either mindfulness or active comparators below this threshold cannot therefore be reliably excluded, and the near-null subgroup estimate should not be read as evidence for equivalence. Meta-regression with *k* = 18 also has limited power for continuous moderators under the residual heterogeneity observed here, so non-significant coefficients should not be interpreted as evidence for the absence of association; the non-significant result for total intervention duration (*p* = 0.062) accordingly requires validation in a larger evidence base. The extrapolation of change-score standard deviations from the MS_error term for a minority of the included studies relies on assumptions (homogeneity of variance and sphericity) that the primary reports rarely substantiate; although the sensitivity analysis excluding these studies indicated limited influence on the pooled estimate, residual conversion error cannot be fully excluded. The concurrent inclusion of state and trait competition-anxiety instruments reflects a conceptual judgement based on their partial construct overlap, but the two classes of instrument differ in temporal reference and in ancillary subscales, and despite a non-significant scale-type moderator they could contribute to residual heterogeneity. The operationalisation of cultural background through Hofstede's individualism–collectivism dimension is one of several defensible approaches, and the findings are conditional on this coding. Finally, the risk-of-bias profile across the included studies (three studies rated high risk, twelve with some concerns, three low risk) introduces methodological variation that the random-effects model partially absorbs but does not eliminate. Uneven cultural distribution−22% of the studies were from China and 22% from Iran (4/18 each)—may limit cross-cultural generalization. Furthermore, there were no purely female samples in the included studies, and eight items were purely male, indicating insufficient representativeness of female athletes and limiting the generalization of the conclusions to female populations. Most studies lacked long-term follow-up (Chen and Meggs, 2021; Wu et al., 2021; Kittler et al., 2022; Jones et al., 2020), and it remains unclear whether the effects can be maintained after the intervention ends. The inclusion of trait and state scales may increase construct heterogeneity, although sensitivity analysis did not find significant differences.

Future research should prioritize conducting more high-quality head-to-head comparative randomized controlled trials (RCTs) of MBI vs. PST/CBT—this represents the most critical evidence gap in the field and is essential for answering the core question of the specific effects of mindfulness. Simultaneously, it should explore long-term maintenance effects and follow-up, introduce objective physiological anxiety indicators to supplement the limitations of self-report scales, conduct multi-center, large-sample RCTs to improve estimation accuracy, and consider network meta-analysis to simultaneously compare the relative effects of multiple psychological interventions (Wang et al., 2025; Yang et al., 2025). Furthermore, the specific contribution of mindfulness may not be reflected in anxiety relief, but rather in psychological processes more directly related to performance, such as cognitive dissociation, acceptance, and flow facilitation—future research could explore this direction in greater depth to more comprehensively understand the unique value of MBI in motor situations.

## Data Availability

The original contributions presented in the study are included in the article/Supplementary material, further inquiries can be directed to the corresponding author.

## Supplementary material

Supplementary Data Sheet 1 Search strategy. Verbatim search strings, dates of last search, and per-database hit counts for all databases searched.
