# Supplementary material for: Mindfulness-based interventions for competitive anxiety in athletes: the moderating role of control type—Preliminary evidence from a systematic review and meta-analysis
Source: Front Psychol. 2026 May 28;17:1832397. doi: 10.3389/fpsyg.2026.1832397 (PMC13253276; doi:10.3389/fpsyg.2026.1832397)
Supplement: Supplementary Data Sheet 1 — Search strategy. Verbatim search strings, dates of last search, and per-database hit counts for all databases searched. [file Data_Sheet_1.docx]

**Supplementary Appendix S1**

**Full Search Strategies for All Seven Databases**

*Manuscript: Mindfulness-Based Interventions for Competitive Anxiety in Athletes: The Moderating Role of Control Type — Preliminary Evidence from a Systematic Review and Meta-Analysis.*

## **Reporting standard**

This appendix reports the verbatim search strategies for the seven databases searched in this systematic review, together with the date of last search and the number of records retrieved from each database, in accordance with PRISMA 2020 (Item #7) and PRISMA-S (2021). All database searches were run from database inception to the dates indicated below. The total of 2,410 records reported in the PRISMA flow diagram (main-text Figure 1) corresponds to the sum of the per-database hit counts in the summary table below. An additional 35 records were identified through supplementary searches, yielding the 2,445 records reported at the identification stage of the PRISMA flow diagram.

## **Summary of database searches**

| **#** | **Database** | **Platform / Provider** | **Date of last search** | **Records retrieved** |
| --- | --- | --- | --- | --- |
| 1 | PubMed | NCBI | 12 October 2025 | 521 |
| 2 | Web of Science Core Collection | Clarivate | 12 October 2025 | 478 |
| 3 | Scopus | Elsevier | 13 October 2025 | 612 |
| 4 | PsycINFO | ProQuest | 13 October 2025 | 287 |
| 5 | SPORTDiscus | EBSCOhost | 14 October 2025 | 234 |
| 6 | Cochrane CENTRAL | Cochrane Library | 14 October 2025 | 68 |
| 7 | CNKI (China National Knowledge Infrastructure) | CNKI | 15 October 2025 | 210 |
|  | **Total** |  |  | **2,410** |

**1. PubMed (NCBI)**

*Date of last search: 12 October 2025*

*Records retrieved: 521*

*Field tags used: [Title/Abstract] (also abbreviated [tiab]). Boolean operators capitalised. Truncation operator: *.*

("mindfulness"[Title/Abstract] OR "mindfulness-based"[Title/Abstract] OR

"MBSR"[Title/Abstract] OR "MAC"[Title/Abstract] OR "MSPE"[Title/Abstract] OR

"MAIC"[Title/Abstract] OR "meditation"[Title/Abstract])

AND

(athlete*[Title/Abstract] OR sport*[Title/Abstract] OR

player*[Title/Abstract] OR competitor*[Title/Abstract])

AND

(anxiety[Title/Abstract] OR anxious[Title/Abstract] OR

"competitive anxiety"[Title/Abstract] OR "sport anxiety"[Title/Abstract])

## **2. Web of Science Core Collection (Clarivate)**

*Date of last search: 12 October 2025*

*Records retrieved: 478*

*Field tag used: TS= (Topic; searches title, abstract, author keywords, and Keywords Plus). Truncation operator: *.*

TS=(mindfulness OR "mindfulness-based" OR MBSR OR MAC OR MSPE OR MAIC OR meditation)

AND

TS=(athlete* OR sport* OR player* OR competitor*)

AND

TS=(anxiety OR anxious OR "competitive anxiety" OR "sport anxiety")

## **3. Scopus (Elsevier)**

*Date of last search: 13 October 2025*

*Records retrieved: 612*

*Field tag used: TITLE-ABS-KEY (searches title, abstract, and keywords). Truncation operator: *.*

TITLE-ABS-KEY(mindfulness OR "mindfulness-based" OR MBSR OR MAC OR MSPE OR MAIC OR meditation)

AND

TITLE-ABS-KEY(athlete* OR sport* OR player* OR competitor*)

AND

TITLE-ABS-KEY(anxiety OR anxious OR "competitive anxiety" OR "sport anxiety")

## **4. PsycINFO (ProQuest)**

*Date of last search: 13 October 2025*

*Records retrieved: 287*

*Field tags used: SU.EXACT (controlled subject heading), TI (title), AB (abstract). Free-text terms combined with thesaurus terms via OR.*

(SU.EXACT("Mindfulness") OR SU.EXACT("Meditation") OR

TI(mindfulness OR "mindfulness-based" OR MBSR OR MAC OR MSPE OR MAIC OR meditation) OR

AB(mindfulness OR "mindfulness-based" OR MBSR OR MAC OR MSPE OR MAIC OR meditation))

AND

(SU.EXACT("Athletes") OR SU.EXACT("Sports") OR

TI(athlete* OR sport* OR player* OR competitor*) OR

AB(athlete* OR sport* OR player* OR competitor*))

AND

(SU.EXACT("Anxiety") OR

TI(anxiety OR anxious OR "competitive anxiety" OR "sport anxiety") OR

AB(anxiety OR anxious OR "competitive anxiety" OR "sport anxiety"))

## **5. SPORTDiscus (EBSCOhost)**

*Date of last search: 14 October 2025*

*Records retrieved: 234*

*Field tags used: DE (subject descriptor), TI (title), AB (abstract). EBSCOhost syntax. Truncation operator: *.*

(DE "Mindfulness" OR DE "Meditation" OR

TI (mindfulness OR "mindfulness-based" OR MBSR OR MAC OR MSPE OR MAIC OR meditation) OR

AB (mindfulness OR "mindfulness-based" OR MBSR OR MAC OR MSPE OR MAIC OR meditation))

AND

(DE "Athletes" OR DE "Sports" OR

TI (athlete* OR sport* OR player* OR competitor*) OR

AB (athlete* OR sport* OR player* OR competitor*))

AND

(DE "Anxiety" OR

TI (anxiety OR anxious OR "competitive anxiety" OR "sport anxiety") OR

AB (anxiety OR anxious OR "competitive anxiety" OR "sport anxiety"))

## **6. Cochrane CENTRAL (Cochrane Library)**

*Date of last search: 14 October 2025*

*Records retrieved: 68*

*Field tag used: :ti,ab,kw (title, abstract, and keywords). Search restricted to the CENTRAL register of trials. Truncation operator: *.*

(mindfulness OR "mindfulness-based" OR MBSR OR MAC OR MSPE OR MAIC OR meditation):ti,ab,kw

AND

(athlete* OR sport* OR player* OR competitor*):ti,ab,kw

AND

(anxiety OR anxious OR "competitive anxiety" OR "sport anxiety"):ti,ab,kw

## **7. CNKI (China National Knowledge Infrastructure)**

*Date of last search: 15 October 2025*

*Records retrieved: 210*

*Search interface: CNKI Advanced Search. Field used: 主题 (Topic, equivalent to title + keyword + abstract). Boolean operators rendered in the Chinese-language interface as AND, OR.*

主题: (正念 OR 正念训练 OR 正念冥想 OR MBSR OR MAC OR MSPE OR MAIC OR 冥想)

AND

主题: (运动员 OR 运动 OR 选手)

AND

主题: (焦虑 OR 竞赛焦虑 OR 比赛焦虑 OR 运动焦虑)

*Equivalent in CNKI Professional Search syntax (SU = Subject/主题):*

SU=('正念' + '正念训练' + '正念冥想' + 'MBSR' + 'MAC' + 'MSPE' + 'MAIC' + '冥想')

AND

SU=('运动员' + '运动' + '选手')

AND

SU=('焦虑' + '竞赛焦虑' + '比赛焦虑' + '运动焦虑')

## **Supplementary searches**

In addition to the database searches above, supplementary searches were conducted by (a) reverse and forward citation tracking of the included studies; (b) hand-searching the reference lists of relevant prior meta-analyses (Wang et al., 2024; Zhang et al., 2025; Li et al., 2025; Si et al., 2024); and (c) grey-literature searching via ProQuest Dissertations & Theses Global. These supplementary searches yielded an additional 35 records, which together with the 2,410 database records produced the 2,445 records reported at the identification stage of the PRISMA flow diagram (main-text Figure 1).
